# Reducing energy system model distortions from unintended storage cycling through variable costs

**DOI:** 10.1016/j.isci.2022.105729

**Published:** 2022-12-07

**Authors:** Maximilian Parzen, Martin Kittel, Daniel Friedrich, Aristides Kiprakis

**Affiliations:** 1 University of Edinburgh, Institute for Energy Systems, Edinburgh EH9 3DW, UK; 2 German Institute for Economic Research, Mohrenstraße 58, 10117 Berlin, Germany; 3 Technical University Berlin, Department of Energy Systems, Einsteinufer 25 (TA 8), 10587 Berlin, Germany

**Keywords:** Energy policy, Energy management, Energy modeling

## Abstract

Energy system models are used for policy decisions and technology designs. If not carefully used, models give implausible outputs and mislead decision-making. One implausible effect is “unintended storage cycling”, which is observable as simultaneous storage charging and discharging. Methods to remove such misleading effects exist, but are computationally inefficient and sometimes ineffective. Through 124 simulations, we find that determining appropriate levels of variable costs depends on the variable cost allocation to certain components and the solver accuracy used for the optimization. For the latter, if the accuracy is set too loosely, the solver prevents the removal of unintended storage cycling. We further provide a list of recommended variable cost model inputs as well as a minimum threshold that can significantly reduce the magnitude and likeliness of unintended storage cycling. Finally, our results suggest that our approach can remove other similar misleading effects such as unintended line cycling or sector cycling.

## Highlights

• Unspecified costs of energy dissipation options may cause excessive storage use
• Observed operational distortions up to 23% for energy storage and 5% for renewables
• Variable costs avoid distortions but depend of solver accuracy and cost allocation
• A list of variable cost suggestions that may alleviate distortions is provided

## Graphical abstract

**Figure:**
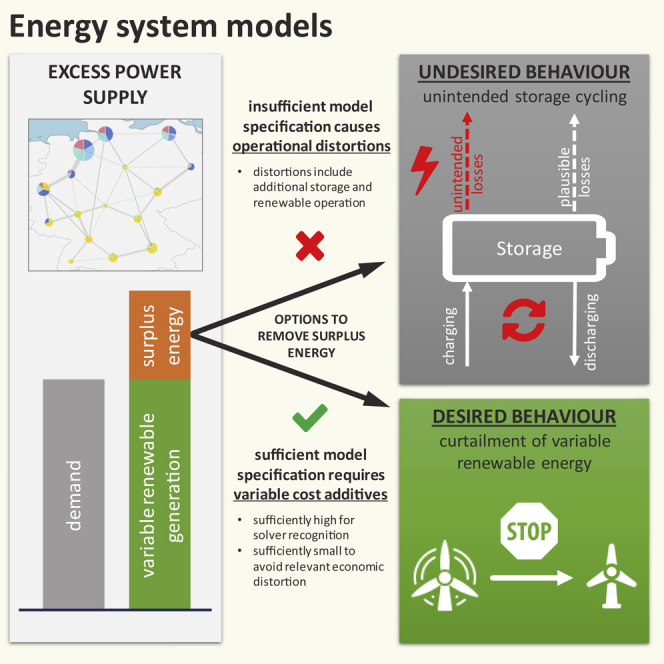

## Teaser

Energy policy; Energy management; Energy Modelling

## Introduction

Energy system models are mathematical models used to investigate possible pathways for decarbonizing our energy systems; in many cases, minimizing total system costs.^1^ They provide insights on optimal dispatch and investment patterns in the short and long term, thus guiding energy technology design decisions^2^ and supporting the decision-making of governments, grid operators, energy system planners, manufacturers, and researchers. However, if not carefully used, such models can mislead decision-making.

**Figure 1:**
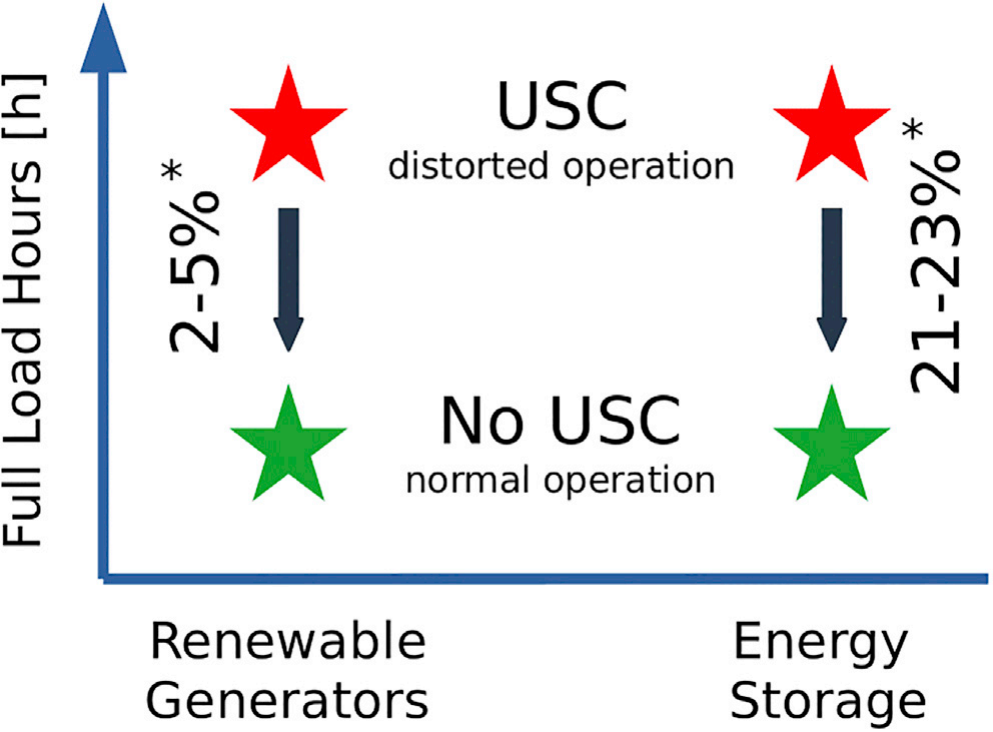
Impact of USC on model outputs Exemplary impact of scenarios with and without USC on storage and renewable generation. The USC effect increases the operation of close to zero variable cost assets. Results from a numerical analysis conducted in this study, marked with an asterisk (∗), show FLH differences of up to 23% for energy storage and 5% for renewable assets in a 100% renewable energy system scenario.

**Figure 2:**
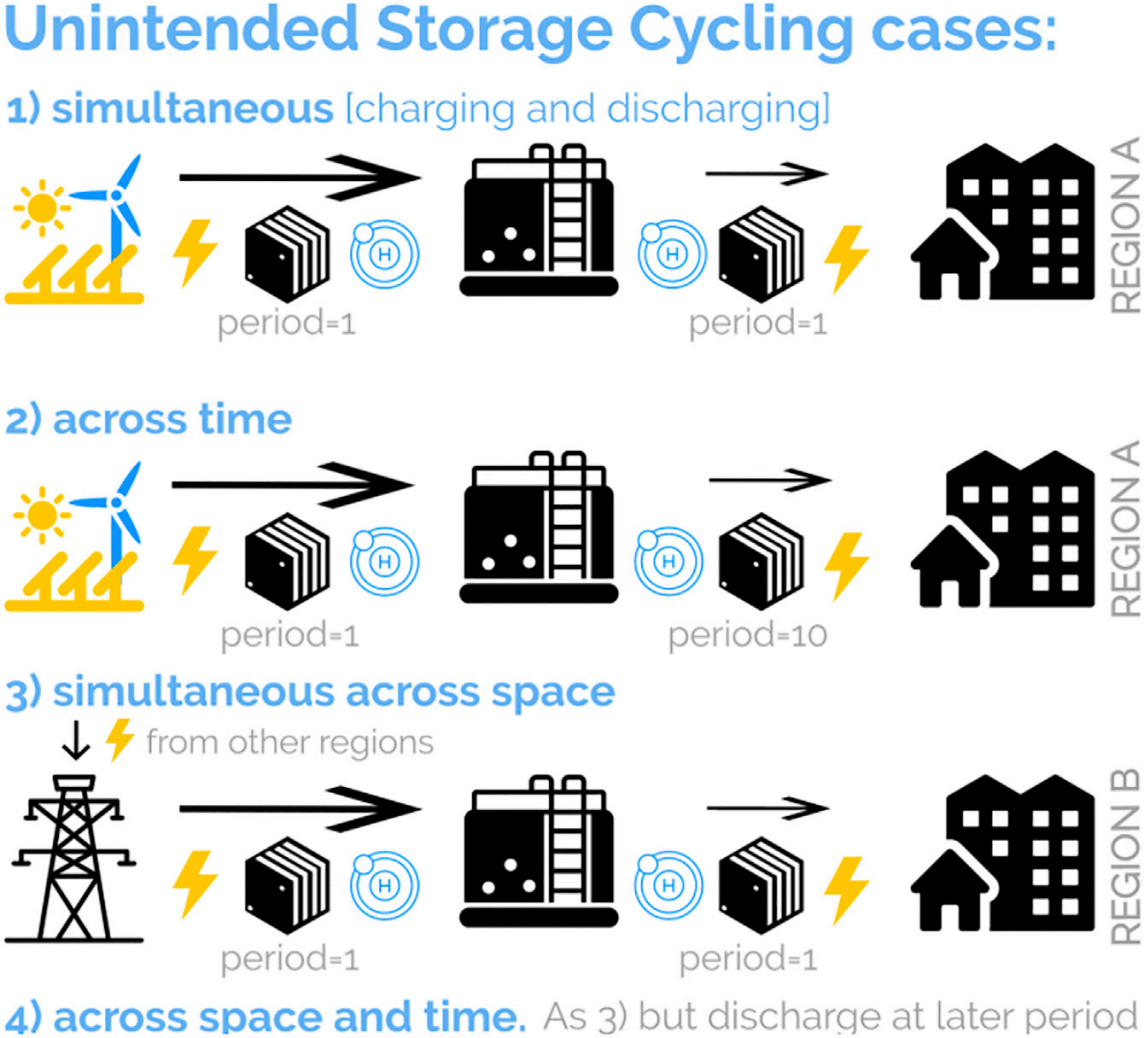
Unintended storage cycling cases for a hydrogen storage example The left stack represents the electrolyzer, while the right stack reflects the fuel cell which converts electricity to hydrogen and vice versa. The arrow size above the storage components reduces to indicate an efficiency drop. Under renewable energy surplus (variable cost = 0), excess energy is removed from the system by USC instead of renewable curtailment. Thereby, USC may occur over space and time if no constraint prohibits this artifact.

One model artifact distorting optimal model results is unintended storage cycling (USC),^3^ which is observed in 12 of 18 well-established energy system models, as reviewed by Kittel and Schill (2022).^3^ The effect impacts storage use. Instead of curtailing variable renewable energy sources (VRE) surplus, the excess electricity is converted, among others, into unintended storage losses by simultaneous charging and discharging of the same storage capacity. The consequence of this behavior is distortions in optimal model outcomes. For example, energy storage or renewable generators may have significantly more full load hours (FLH) in a scenario with, compared to one without USC (Figure 1), signaling deceptively more intensive operation. Furthermore, USC is technically infeasible for some storage technologies, e.g. single lithium-ion batteries, that can either charge or discharge but not both simultaneously. Hence, the effect urges its removal. USC may also manifest across space and time (Figure 2). For instance, USC across space may occur in multi-regional model settings through simultaneous charging in one region and discharging in another region for the sole purpose of dissipating surplus renewable energy instead of curtailing it, notably in the absence of transmission costs. Similarly, USC across time represents unintended simultaneous charging and discharging cycles across multiple periods.^3^ The fact that USC is not limited to one point in space and time aligns with the non-guaranteed operational uniqueness in scenarios with multiple storage assets.^4^

USC is not the only such misleading effect. There is a group of related effects classified under the term unintended energy losses, which arise in a cyclic manner.^3^ Unintended, because they distort optimization results. Cyclic, as they occur in the energy systems wherever efficiency losses are present between energy components that cycle energy by charging and discharging, sending and receiving, or converting and re-converting operations.

The literature on unintended energy losses is limited. State-of-the-art guidance on best-practice energy system modeling probably unintentionally ignores these artifacts,^5^^,^^6^ while others do this intentionally.^7^ Attempts to remove unintended energy losses exist. For instance, an intuitive approach is to prohibit simultaneous charging and discharging by introducing a binary variable. This binary variable can then represent two mutually exclusive storage operational modes: charging or discharging, with only one being possible at the time. Introducing a binary variable requires reformulating the optimization from a linear to a mixed-integer problem.^8^ This reformulated problem is not only harder to solve and require more computations but also does not guarantee the full USC removal due to its occurrence across space and time. Differently,^9^ penalizes active power losses in the objective; however, as described in,^8^ this approach can distort model outputs under heavy load conditions. This is probably the case because one uniform penalty applied to all technologies that experience losses does not recognize any operational order.

While the above literature provides solutions on USC arising in models that abstain from binding renewable energy targets, Kittel and Schill (2022)^3^ investigate USC arising in models that are constrained by a binding renewable energy target. In these models, USC causes an increase of VRE generation, which can be realized without additional renewable capacity installations. Thus, the renewable energy target can be achieved with less VRE capacity at lower costs. Yet, it does not serve demand, which requires additional generation from other dispatchable technologies. Here, USC flaws both optimal dispatch and investment decisions. To remove USC, the renewable energy constraint must include these unintended energy losses, preventing the cost-minimizing conversion of intended VRE curtailment into unintended energy losses.

However, the novel solution presented in Kittel and Schill^3^ is not applicable to model formulations *without* binding renewable targets,^10^^,^^11^^,^^12^^,^^13^ which are more frequently used than models with binding renewable targets.^3^ In models without binding renewable targets, USC is caused by a different mechanism: It may arise if one or multiple options to dissipate unused energy from the system are available and come at e.g. zero cost. Given such a parameterization, the optimization becomes indifferent with regard to the use of any energy dissipation options. VRE surplus energy can either be curtailed or erased from the system via unintended energy losses from USC, distorting optimal dispatch results (see conclusion and removing unintended storage cycling by variable cost additives). Thus far, the removal of unintended storage cycling in energy models without binding renewable constraint is unexplored.

**Figure 3:**
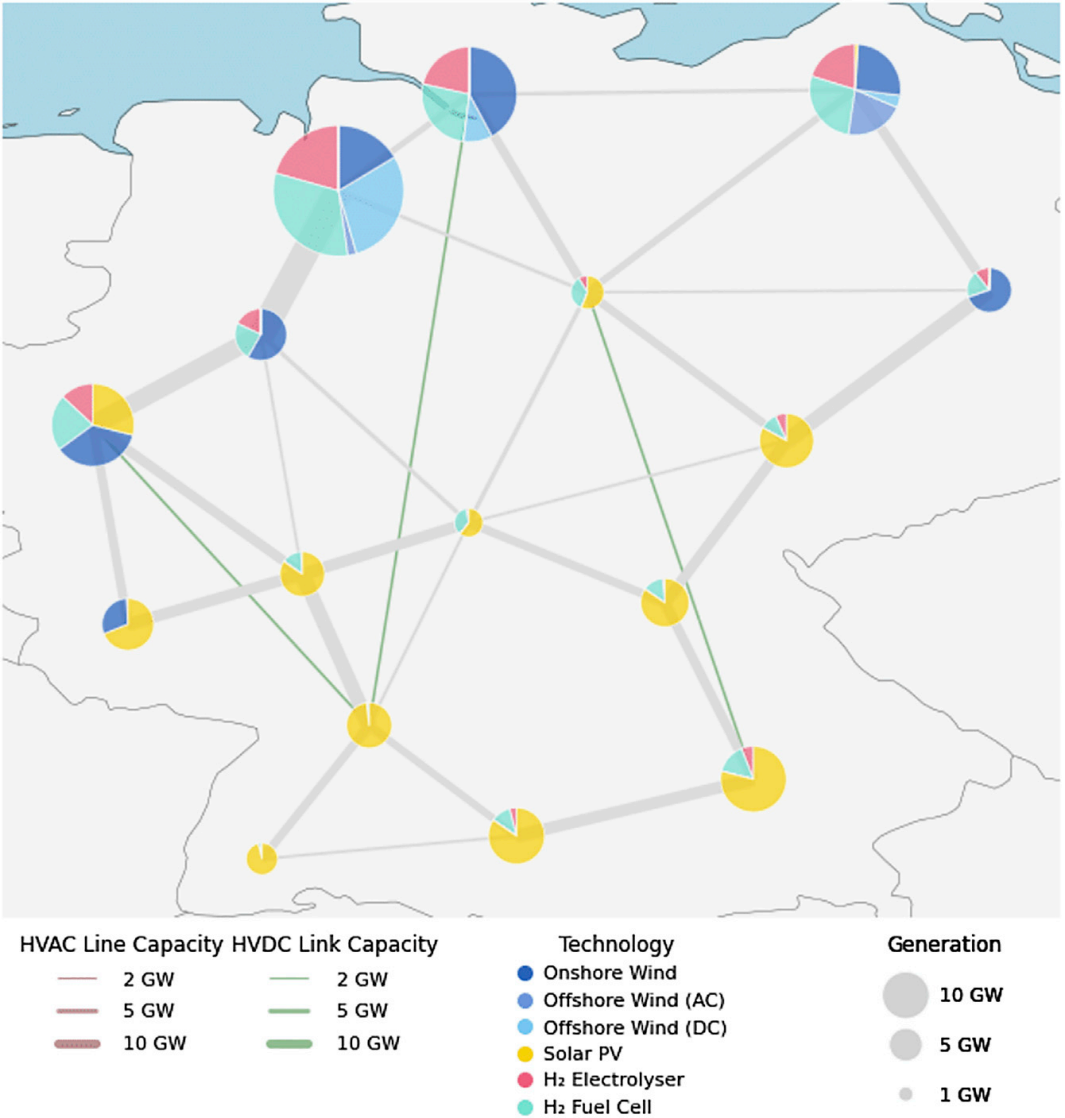
Illustration of model output It shows an example model scenario with optimized generation and storage capacities in Germany for a 100% GHG emission reduction case.

This paper addresses this gap by contributing to the existing literature in several aspects: Firstly, we provide a method for the removal of USC in linear models *without* binding renewable energy targets. We show that the removal can be achieved by a deliberate setting of variable costs of affected system components. Since variable costs penalize not only USC but also the operation of affected system components, these must be carefully chosen. Unlike MILP-based approaches, this solution keeps the problem formulation linear, making it more effective and efficient. Secondly, we mathematically formalize how the simultaneous charging and discharging case of USC occurs. Thirdly, we explore the impact of USC and its removal on operational and investment decisions as well as total system cost in a decarbonized German energy system model (Figure 3) with parameter sweeps for the variable cost of technologies (Table 1). To this end, we demonstrate how variable costs, their magnitude, and allocation can remove USC, while also giving new insights on the role of the solver accuracy. Finally, we provide a reviewed list of variable cost inputs, which may guide the removal of USC in other energy models.

## Results and discussion

In the following subsections, we introduce how to remove USC as well as investigate the impact of USC and its removal on model outcomes such as optimal dispatch, installed capacity, and total system cost. The stylized model formulation, its numerical implementation, and the experimental setup are described in the conclusion.

### Removing unintended storage cycling by variable cost additives

We define variable cost additives as not necessarily true observed variable costs, but more generally as assumed additional costs components.

Suppose an optimization problem, such provided in the model formulation Conclusion, finds a least-cost total system architecture. Then, the system operation may adapt any value as long as it does not lead to more cost (left side of Equations 1 and 2) and does not break constraints such as demand is equal to supply (described in 4).

Furthermore, suppose the energy system contains a renewable energy surplus at a time step, while variable operational cost $o_{i,s/r}$ of storage and renewables are assumed to be zero, then:

(Equation 1) $$0=\underset{0}{\underset{︸}{o_{i,r}}}\cdot g_{i,r,t}^{*}+\underset{0}{\underset{︸}{o_{i,s}^{\text{+}}}}\cdot h_{i,s,t}^{*,+}+\underset{0}{\underset{︸}{o_{i,s}^{\text{−}}}}\cdot h_{i,s,t}^{*,-}+\underset{0}{\underset{︸}{o_{i,s}^{store}}}\cdot △e_{i,s,t}^{*}$$

These zero costs may lead to a situation where surplus generation is fed into the grid rather than curtailed. However, to guarantee the energy balance, extra surplus generation needs to be dissipated by USC (indicated by ∗), which leads to higher storage usage.

In contrast, in case costs exist for either generation or storage operation,

(Equation 2) $$0=o_{i,r}\cdot \underset{0}{\underset{︸}{g_{i,r,t}}}+o_{i,s}^{\text{+}}\cdot \underset{0}{\underset{︸}{h_{i,s,t}^{\text{+}}}}+o_{i,s}^{\text{−}}\cdot \underset{0}{\underset{︸}{h_{i,s,t}^{\text{−}}}}+o_{i,s}^{store}\cdot \underset{0}{\underset{︸}{△e_{i,s,t}}}$$

every additional operation of variable renewable generators or storage is prevented in the first place, thus avoiding USC.

Equations 1 and 2 illustrate that a system with USC (indicated by ∗) has components with higher operating hours than one without,

(Equation 3) $$\sum_{t=1}^{T}g_{i,r,t}^{*}\geq \sum_{t=1}^{T}g_{i,r,t}$$

(Equation 4) $$\sum_{t=1}^{T}h_{i,s,t}^{*,+/-}\geq \sum_{t=1}^{T}h_{i,s,t}^{+/-}$$

(Equation 5) $$\sum_{t=1}^{T}e_{i,s,t}^{*}\geq \sum_{t=1}^{T}e_{i,s,t}$$

caused by energy dissipation through excessive storage use rather than curtailing renewable surplus.

In summary, to remove USC, a situation with USC must become more expensive than one without because, fundamentally, the objective function aims to minimize cost. One approach is to add variable cost $o_{i,r}>0$ to the generation dispatch. Another is to add variable costs $o_{i,s}>0$ to any or all energy storage components, such as charger, store, or discharger. All such variable cost additives penalize any extra operation of generators or storage units caused by USC energy dissipation, even across space and time. Nevertheless, since variable costs penalize not only USC but also the operation of these units, these must be carefully chosen.

### Effects on operational optimization

**Figure 4:**
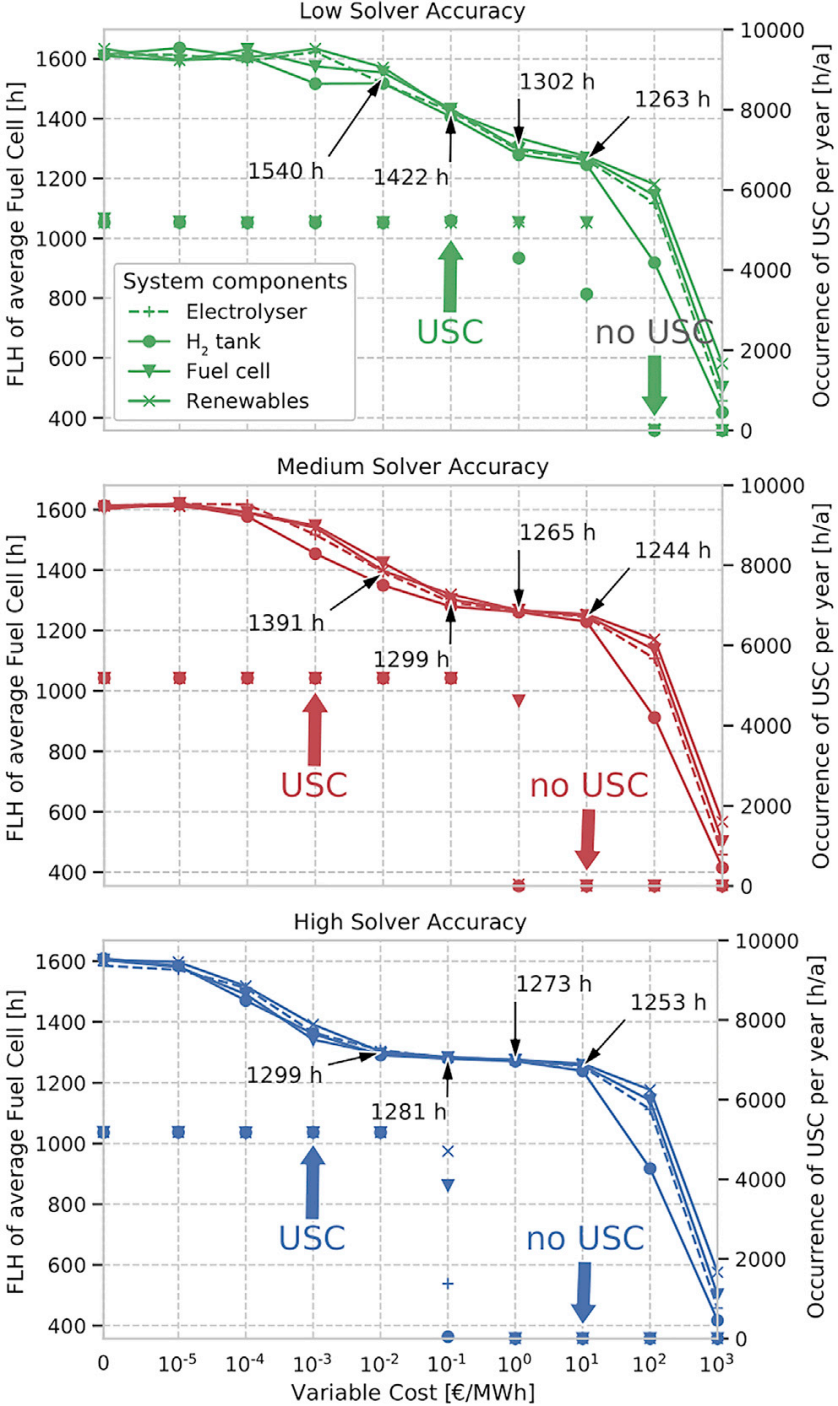
Analysis of USC for different model scenarios FLH of the H2 fuel cell (lines, left y axis) and occurrences of USC (scatterplots, right y axis) for three levels of solver accuracy (low, medium, high from top to bottom) across different levels of variable cost additives for renewable generators or H2 storage components (x axis). FLH are averaged across all nodes in Germany. Per scenario, the variable cost additives are added to only one component, while no variable costs accrue for all others.

Figure 4 illustrates the number of hours with USC in the system (scatterplots, right y axis). It further shows FLH of the hydrogen (H2) fuel cell for varying variable costs of the renewable generators or H2 storage components (lines, left y axis). The approach to count the USC occurrence is given in conclusion.

Adding variable cost to any class of storage components or all renewables can successfully remove USC beyond a certain threshold that depends on the solver accuracy. The observed occurrence of USC spatially averaged over all modeled nodes with variable cost below $10^{-3}$ is roughly 5200, regardless the level of solver accuracy. Note that for the determination of the USC occurrences, any simultaneous charging and discharging below the energy value of 1 MWh is not counted as USC to ensure that only significant USC energy volumes are considered. Otherwise, USC would occur in almost every time step of every scenario with marginal energy volumes, which may be caused by the solver tolerance and the non-uniqueness of the optimal operation of storage assets.^4^

More importantly, the USC energy volume decreases for increasing cost additives, irrespective of the USC occurrence counting method. This decrease is illustrated by the FLH curves in Figure 4. In general, the FLH curves reveal that adding variable costs affects the operation of the storage system. The decline in FLH as the variable cost increase is due to two overlapping effects, indicating a trade-off between the removal of USC and an undistorted operation of the storage system. For the lower range of the investigated variable cost additive scenarios, the reduction of the USC energy volume is the prime driver of the FLH decline. In contrast, very high cost additives render the storage system’s operation less economically viable, strongly decreasing its optimal use. In the medium-to-high range of the cost additives, both USC is prevented and storage operation remains largely constant and undistorted, indicated by the plateau of the FLH curves.

In our stylized setting at medium solver accuracy—which refers to the PyPSA-Eur default values^14^—USC is fully removed in any considered scenario with a variable cost threshold of at least 10 €/MWh or 1 ct/kWh. While for scenarios with a variable cost additive of 1 €/MWh USC still occurs, the USC energy distortions are only marginal with a slight increase of the fuel cell’s FLH of 21 h compared to a cost additive scenario with 10 €/MWh (1265–1244 h). Hence, a variable cost additive of slightly above 1 €/MWh (or 0.1 ct/kWh) is likely to prevent USC distortions in our case study.

The very cost additive threshold that removes USC depends on the level of solver accuracy. In the case of low solver accuracy, the threshold is relatively high at 100 €/MWh. However, FLH curves hardly stabilize in a plateau, which would indicate that storage operation remains unaffected. This makes it difficult to identify the optimal cost additives that prevent USC. Furthermore, such a high variable cost additive level is implausible for VRE or storage components. For medium and high solver accuracy, FLH curves form a plateau, with the lower end at 10 and 1 €/MWh, respectively. Note that we discretely increment the variable cost additives by one order of magnitude. The true underlying thresholds, defined by the minimum variable costs that remove all USC, may be in between these increments.

The observed impacts of USC on the operation are extreme by design of this study. The operational distortions are amplified through the exclusion of dispatchable renewable and conventional generators, such as biomass, nuclear, or green gas. Including such dispatchable generators may decrease the USC energy distortions in many energy models, as this would introduce additional variable costs that reduce the impact of USC (see removing unintended storage cycling by variable cost additives). Nevertheless, this study also reveals that assuming no variable cost for generators or storage technologies, as done in multiple if not most energy system modeling studies,^3^^,^^15^^,^^16^^,^^17^^,^^18^ risks unintended operational distortions. We investigate if these distortions, as well as variable cost additives, also impact the investment optimization in the next section.

### Effects on investment optimization

**Figure 5:**
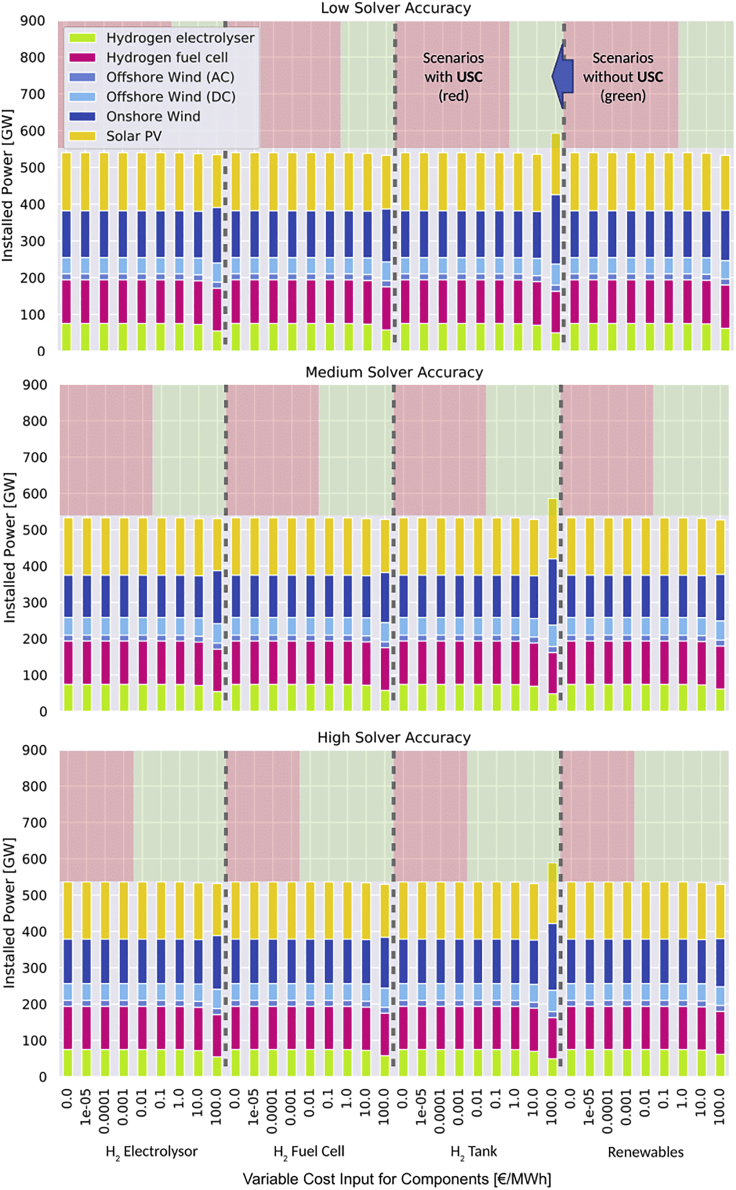
Installed capacity of all generation and storage assets for different variable cost additive scenarios In scenarios in red USC arises, while in the green the effect is prevented. We omit illustrating results from the scenarios using variable costs additives of 1000 €/MWh to keep Figures 5 and 6 consistent and readable.

Figure 5 illustrates the optimized generation and storage capacity of all modeled scenarios. The optimal installed capacity is much more robust to cost additives than optimal operation. At additives of 1 €/MWh, which is sufficient to avoid USC occurrence, optimal capacity results remain unaffected. Only for very high additives of 10–100 €/MWh the optimal installation is affected.

In scenarios with very high VRE variable cost additives, wind power is used more, while both photovoltaic (PV) and storage are used less. This is due to the more stable generation pattern of wind power compared to solar PV which requires more storage to smooth its diurnal generation profile. Additionally, even though variable costs are added only to generator operations, the use of storage becomes more expensive as storage efficiency losses multiply VRE generation cost. For instance, at an electricity price of 100 €/MWh and a round-trip efficiency of 25%, discharging 1 MWh comes at energy procurement costs of 4 MWh × 100 €/MWh (4 MWh must be charged to generate 1 MWh of storage output). This multiplication effect of generation costs in energy storage components would be less of an issue if generation had lower costs. For instance, consider variable generation costs of 0 €/MWh for the same efficiency as before. The effective operation costs of charging and discharging were zero—yet, causing USC.

For plausible variable cost additives, USC does not impact optimal investment decisions. This is one major difference to USC arising in energy models with a renewable energy constraint, where the artifact causes complex distortions of optimal investments.^3^

### Effects on total system costs

**Figure 6:**
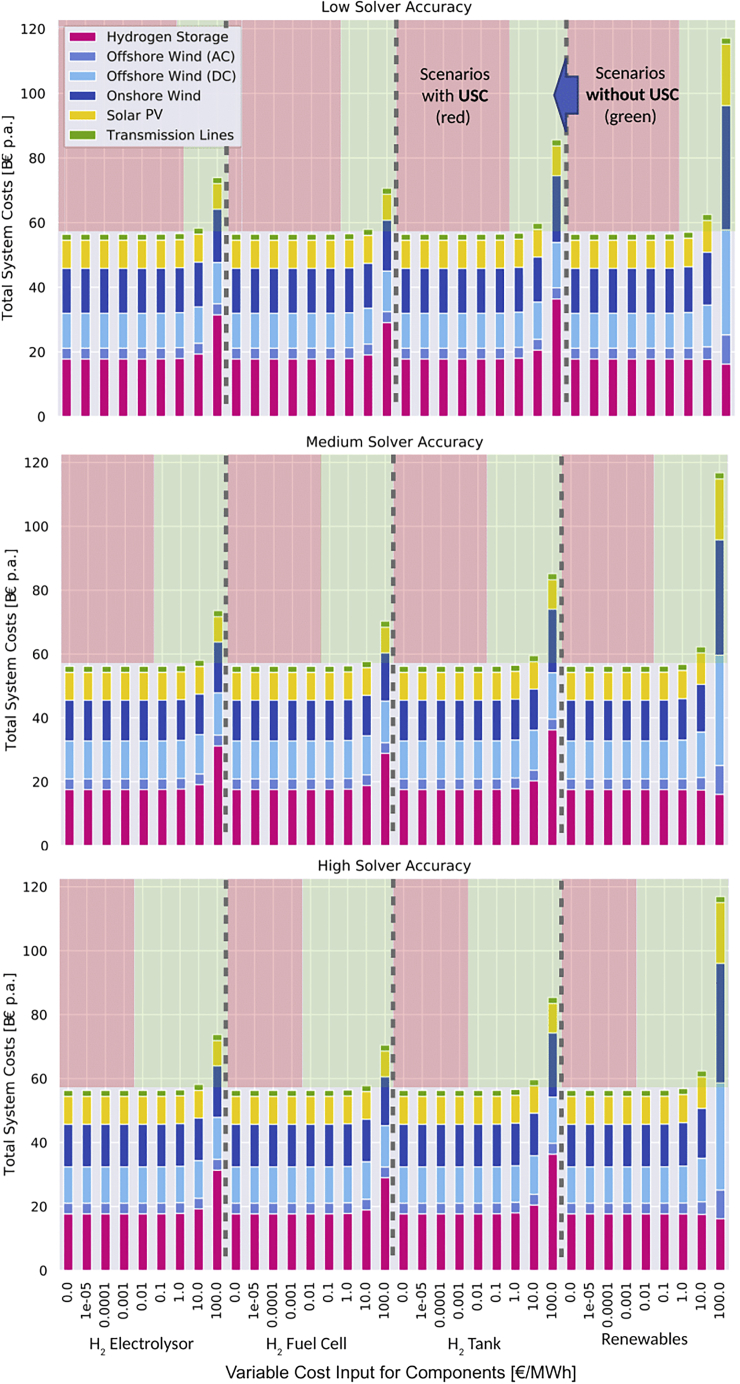
Total system costs for different variable cost additive scenarios In scenarios in red USC arises, while in the green the effect is prevented. Costs of the hydrogen storage consist of electrolyzer, fuel cell, and H2 storage tank. We omit illustrating total system costs from the scenarios using variable costs additives of 1000 €/MWh for readability.

The total system costs consist of operational and investment cost and is a key parameter to assess the wider energy system. Figure 6 shows the total system cost results for all optimization runs stacked by system components. It reveals that scenarios with USC and applied USC removal strategies have only negligible impact on the total system costs unless the variable costs are set too high (above or equal to 10 €/MWh). Depending on the technology for which the variable costs are added, a significant cost increase can be detected due to the extra operational cost that needs to be covered. Again, the assumed values, for instance, of 100 €/MWh for the dispatch of all included renewables, might not be realistic but illustrate the impact of mistakenly choosing the wrong values.

### Variable costs suggestions to alleviate unintended storage cycling

Our results suggest that variable costs should be carefully set for all assets to guarantee the removal of USC while avoiding any distortion of optimal investment and dispatch decisions. In our stylized setting, the lower end of the plateau of the FLH curves in Figure 4 indicates the optimal threshold for an appropriate cost additive. Values below this threshold do not prevent USC, while values above this threshold can lead to investment and operational distortions.

Providing variable costs above a minimum threshold is key to avoid USC in models without binding renewable targets, even for technologies with zero or near-zero variable costs. For instance, if a solar PV plant is given no or too small variable costs, then solvers cannot recognize them. Consequently, these values should be replaced by the minimum value depending on the solver accuracy and used solver. In our stylized setting of PyPSA-Eur, this minimum value is 1 €/MWh at the used default Gurobi solver settings of ($BarConvTol=1e^{-5}$, $FeasibilityTol=1e^{-6}$), which remove most USC effects. While 1 €/MWh or 0.1 ct/kWh may be considered as relatively high, our results in Figure 4, Figure 5, Figure 6, 5, and 6 show that these little variable costs neither affect the investment nor the operational costs significantly, making it a threshold candidate to alleviate large USC distortions.

From Figure 4, it is observed that there are multiple options to remove USC. First, setting minimal variable cost only for one storage component, such as charger, store, or discharger for all storage technologies in all regions across the network. Second, imposing minimal variable costs to all VRE generation types at all locations (see Equations (Equation 1), (Equation 2)). Third, one can also combine the first and second point and impose minimal variable costs to all storage and generator assets. With likely negligible effects on computational time and total system cost distortion, we recommend the third option as it is most consistent and provide additional redundancy to remove USC.

The fact that some technologies are assumed with zero costs for energy dispatch (see 2) could be reconsidered. The concept of *variable cost for lifetime reductions* may justify cost assumptions for non-zero dispatch generators or storage components. Suppose a wind turbine operates 100% and another one only 50% of all hours of a year. Furthermore, both turbines are equally maintained by contract-based operation and maintenance service providers (often annualized as fix-costs). However, the twice as much operating turbine is likely to experience, on average, earlier signs of fatigue in the mechanical structures and power electronics.^19^^,^^20^ As a result, the turbine with more operating hours was indeed experiencing costs, namely variable costs for lifetime reductions that are associated with the reduced technical lifetime or extra required operation and maintenance. Such variable cost for lifetime reductions cost may be not trivial and vary across technologies. For instance, in the case of thermal-based processes like steam turbines in concentrated solar power plants, a steady rather than fluctuating operation is preferred. This reduces thermal stresses that otherwise shorten the plant lifetime.^21^ In summary, even though associating variable cost to the technical lifetime is not trivial, these costs are likely to appear in the energy systems often in addition to other variable costs, e.g. parasitic loads and water consumption. These variable costs make it likely to argue for variable costs greater than zero that might even reach the minimum variable cost of 1€/MWh required to significantly remove the risk of unintended storage cycling.

In Table 2, we provide a comprehensive list of variable costs suggestions, including a minimum threshold for technologies that are considered near-zero or zero variable cost devices.^22^^,^^23^ Replacing the zero or near-zero variable costs by the threshold often insignificantly increase computations since relatively few additional variables need to be optimized. In general, these tabulated values contain large uncertainties since they are not provided in great detail in the literature.^24^ The suggested threshold of 1 €/MWh has to be taken with caution as it may not apply to all energy models. The threshold is recommended for a specific modeling tool, namely PyPSA-Eur, while using the Gurobi solver with default accuracy of $BarConvTol=1e^{-5}$, $FeasibilityTol=1e^{-6}$. If the model formulation, the solver, or the solver parameters differ, then this suggestion may no longer be valid. So while the ideal threshold may require quantification for each model parameterization separately, the values in Table 2 could serve as a default starting point for the identification of an appropriate cost additive.

**Table 2:** Variable O&M cost suggestions

Technology	variable cost [€/MWh]	Source
onshore wind	1.4	DEA^26^
offshore wind	2.7	DEA^26^
PV	$0\to 1^{*}$	Clauser & Ewert^30^
CSP a	2.9	Clauser & Ewert^30^
CSP + Storage	4	Clauser & Ewert^30^
Biomass	6.7^a^	Clauser & Ewert^30^
Tidal	3.1^d^	-.
Wave	3.0^d^	-.
Geothermal	5.6	Clauser & Ewert^30^
run of river	3.6^a^^,^^b^	EIA^31^
hydroelectric dams	3.6^a^^,^^b^	EIA^31^
pump-hydro storage	3.6^a^^,^^b^	EIA^31^
battery inverter	6.8^a^^,^^c^	EIA^31^
battery storage	13.5^a^^,^^c^	EIA^31^
hydrogen electrolyzer	3^e^	Glenk & Reichelstein^32^
hydrogen storage tank^f^	$0^{f}\to 1^{*}$	DEA^26^
hydrogen fuel cell^f^	$0^{f}\to 1^{*}$	DEA^26^

The variable O&M cost are for a set of renewable generators and storage technologies in energy models based on 2030 data. The O&M costs exclude fuel cost, e.g. for biomass.

^∗^ Reported below 1€/MWh, but set to 1€/MWh to avoid USC.

a Interpolated between 2020 and 2035.

b Aggregated as hydroelectric devices by EIA.

c Assumption of cost split: 2/3 store and 1/3 inverter.

d Assumed similar to offshore wind. Lack of alternative data.^33^

e Required conversion with hydrogen energy density of 33.3kg/MWh

f DEA reports zero variable costs for the whole hydrogen storage system.

### Limitations of the study

In our case study, the minimum variable cost threshold of 1 €/MWh (or 0.1 ct/kWh) removes all significant USC effects at default Gurobi solver accuracy settings without impacting the overall optimization significantly. However, this threshold cannot be generalized to all other energy models. Instead, the efficacy needs to be tested for each model, solver, and parameterization. This can be done by checking for simultaneous charging and discharging while maintaining an appropriate level of solver accuracy.

### Conclusion

Reliable energy model results are essential for planning optimal pathways for the energy transition. However, in energy models without binding renewable energy targets, USC can distort operational results of optimized energy systems, while it keeps investment results unaffected. This means that policy questions related to optimal capacity expansion can be answered while disregarding the occurrence of USC. However, when any operational signals are discussed such for technology evaluations, then assessing USC is important. In our case study, the modeling artifact significantly increased the FLH of storage and renewable assets of up to 23%, potentially misleading decision-makers. Since USC is technically infeasible for some storage technologies, e.g. single lithium-ion batteries, and can lead to significant operational distortions, it should be removed.

We show that setting an appropriate level of variable costs is capable of removing USC, while keeping the problem formulation linear and convex. However, determining this level is not trivial. The optimization solver may not recognize too low variable costs, which then does not guarantee the removal of USC. Hence, we recommend setting minimum variable costs at a certain threshold that depends on the solver’s accuracy and tolerance. Very high variable cost, on the other side, may prevent USC but can also significantly distort the relative cost ratio of available generation and balancing technologies. As a consequence, optimal investment and dispatch decisions may be flawed. To avoid such model distortions, it is essential to set the variable cost carefully and as accurately as possible.

We provide a selection of recommended variable costs for a set of storage and renewable generation technologies extracted from the literature. These values include the identified threshold of 1 €/MWh (or 0.1 ct/kWh) as a minimum level to remove all significant USC. While we did not apply the minimum threshold to all storage and renewable generation technologies at the same time, we already prove in Figure 4, Figure 5, Figure 6, 5, and 6 that the threshold sufficiently reduces USC distortions without changing the optimization result much when applied for either one of the storage components or all generators. Thus, the values in Table 2 should be taken as baseline. The suggestions may also serve as a starting point for USC tests in other model applications following the rule-of-thumb: near-zero variable costs additives lower the risk of significant USC distortions compared to assuming no variable costs at all.

Future work should analyze the removal of other forms of unintended energy losses beyond USC by setting appropriate variable costs. For instance, unintended line cycling manifests by simultaneous sending and receiving of electricity through power lines in the distribution and transmission grid^3^^,^^25^; and sector-coupled cycling, e.g., by electric energy that is converted to heat by boilers and re-electrified at the same time with organic Rankine cycle plants. These unintended energy losses may originate from the same issue, namely that missing operational costs make a cost-minimizing energy model indifferent between possible options to lose unused energy either by cyclic dissipation or VRE curtailment. Furthermore, while this study investigates USC in energy models triggered by insufficiently specified cost assumptions, USC also arises when using additional constraints, e.g., binding renewable energy targets.^3^ Constraint-based unintended energy losses are not yet fully explored and merit future research. Finally, USC can be caused by charging and discharging at the same time or across space and time. Throughout the paper, we only detect USC caused by simultaneous charging and discharging. We do this being aware that variable cost additives penalize any extra operation of generators or storage units caused by USC energy dissipation, even across space and time. However, to prove this, future work should explore USC detection methods across space and time.

Other future work can also investigate alternative methods to remove USC. A two-step optimization approach, which has yet to be discussed in the literature, may be able to remove USC while not risking to distort the optimization results. The first step minimizes the total system costs in an investment and dispatch co-optimization, which results in a solution with unintended storage losses. The second step adds the objective value from the first step as a side constraint in a dispatch optimization which minimizes the operational losses or maximizes the generator curtailments in the system to eliminate unintended storage losses. While solutions times between the variable cost and the two-step optimization approach are likely to be in the similar range, future work can compare these methods in more detail.

## STAR★Methods

### Key resources table

**Table:** 

REAGENT or RESOURCE	SOURCE	IDENTIFIER
**Software and algorithms**		

PyPSA-Eur	Hörsch et al.^34^	https://doi.org/10.1016/j.esr.2018.08.012
HiGHS solver	Huangfu and Hall^35^	https://doi.org/10.1007/s12532-017-0130-5

### Resource availability

#### Lead contact

Further information and requests for resources and reagents should be directed to and will be fulfilled by the lead contact, Maximilian Parzen max.parzen@ed.ac.uk.

#### Materials availability

Not applicable.

### Method details

#### Model formulation

The occurrence of USC depends on the model formulation.^3^ This section only introduces a general formulation of investment and dispatch optimization problems for energy system models that abstain from binding renewable energy target constraints. The specific model use and formulation for the demonstration are described in Section 4.0.2.

The objective of such models is to minimise the total system costs, comprised of annualised capital and operational expenditures. Capital expenditures include capacity-related, long-term investment costs c at location i for generator capacity $G_{i,r}$ of technology r, storage energy capacity $H_{i,s}^{store}$, charging capacity $H_{i,s}^{\text{+}}$, and discharging capacity $H_{i,s}^{\text{−}}$ of technology s and transmission line capacity $F_{l}$. Operational expenditures include energy-related variable cost o for generation $g_{i,r,t}$, storage charging $h_{i,r,t}^{+}$, discharging . $h_{i,r,t}^{\text{−}}$., as well as the energy-level related storage cost $e_{i,s,t}$. Thereby, the operation depends on the time steps t that are weighted by duration $w_{t}$ that sums up to one year $\sum_{t=1}^{T}w_{t}=365days*24h=8760h$. The model assumes linear cost functions, price-inelastic demand, perfect foresight, and a perfect energy-only market. In the long-term market equilibrium, these assumptions yield zero profits for all market participants.

(Equation 6) $$\begin{array}{l} \min_{G,H,F,g,h,e}\;\left(Total\;System\;Cost\right)=\\ \min_{G,H,F,g,h,e}\;[\sum_{i,r}\left(c_{i,r}\cdot G_{i,r}\right)+\sum_{l}\left(c_{l}\cdot F_{l}\right)+\sum_{i,s}\left(c_{i,s}^{store}\cdot H_{i,s}^{store}+c_{i,s}^{\text{−}}\cdot H_{i,s}^{\text{−}}+c_{i,s}^{\text{+}}\cdot H_{i,s}^{\text{+}}\right)+\sum_{i,r,t}\left(o_{i,r}\cdot g_{i,r,t}\cdot w_{t}\right)+\sum_{i,s,t}\left(\left(o_{i,s}^{\text{+}}\cdot h_{i,s,t}^{\text{+}}+o_{i,s}^{\text{−}}\cdot h_{i,s,t}^{\text{−}}\right)\cdot w_{t}\right)\\ +\sum_{i,s,t}\left(o_{i,s}^{store}\cdot e_{i,s,t}\cdot w_{t}\right)] \end{array}$$

The objective function can be subject to multiple linear constraints. An example is laid out in more detail in,^34^^,^^35^ leading to a convex linear program. A convex linearly formulated problem has a unique objective value with sometimes multiple non-unique operational solutions. For instance, when various ways exist to distribute the storage and generation operation temporally and spatially, in the absence of spatial limitations (i.e., available transmission capacity) or temporal limitations (i.e., available energy store capacity) for consecutive time steps. This is also the reason why USC can occur over space and time, as mentioned in the introduction, arising in non-unique patterns as long as it does not break any constraint or lead to higher costs.

Further, constraints in the energy model include:

• nodal power balance constraints guaranteeing that supply equals demand at all times,
• linearised power flow constraints modeling the physicality of power transmission (Kirchhoff’s Voltage and Current Law),
• hourly solar and wind resource availability constraints limiting the renewable generation potential based on reanalysis weather data,
• land-use constraints, restricting the renewable capacity expansion based on environmental protection areas, land use coverage, and distance criteria, and finally,
• emission constraints introducing a limit of $CO_{2}$ equivalent GHG emissions.

Storage charging $h_{i,s,t}^{\text{+}}$ and discharging $h_{i,s,t}^{\text{−}}$ are both positive variables and limited by the installed capacity $H_{i,s,t}^{\text{+}}$ and $H_{i,s,t}^{\text{−}}$.

(Equation 7) $$0\leq h_{i,s,t}^{\text{+}}\leq H_{i,s}^{\text{+}}\forall i,s,t$$

(Equation 8) $$0\leq h_{i,s,t}^{\text{−}}\leq H_{i,s}^{\text{−}}\forall i,s,t$$

This formulation keeps the feasible solution space convex.

The storage energy level, $e_{i,s,t}$, is the result of a balance between energy inflow, outflow, and self-consumption. Additional to directed charging and discharging, with its respective efficiencies $\eta_{i,s,+}$ and $\eta_{i,s,-}$, natural inflow $h_{i,s,t}^{inflow}$, spillage $h_{i,s,t}^{spillage}$, and standing storage losses that reduce the storage energy content of the previous time step by a factor of $\eta_{i,s,+}$ are considered.

(Equation 9) $$e_{i,s,t}=\eta_{i,s,+}\cdot e_{i,s,t-1}+\eta_{i,s,+}\cdot w_{t}\cdot h_{i,s,t}^{\text{+}}-\eta_{i,s,-}^{-1}\cdot w_{t}\cdot h_{i,s,t}^{\text{−}}\;+w_{t}\cdot h_{i,s,t}^{inflow}-w_{t}\cdot h_{i,s,t}^{spillage}\;\forall i,s,t$$

The amount of energy that can be stored is limited by the energy capacity of the installed store unit $H_{i,s}^{store}$ MWh., which allows independent storage component scaling.

(Equation 10) $$0\leq e_{i,s,t}\leq H_{i,s}^{store}\;\forall i,s,t$$

For fixing the storage technology design, a technology-specific energy to discharging power ratio ${\bar{T}}_{s}$ can be multiplied by the capacity of the discharging unit $H_{i,s}^{\text{−}}$

(Equation 11) $$0\leq e_{i,s,t}\leq {\bar{T}}_{s}\cdot H_{i,s}^{\text{−}}\;\forall i,s,t$$

to define the upper energy limit per installed storage.

Finally, energy storage units are assumed to be cyclic, i.e., the state of charge at the first and last period of the optimization period T (i.e. 1 year) must be equal:

(Equation 12) $$e_{i,s,0}=e_{i,s,T}\;\forall i,s$$

This cyclic definition is not mandatory but helps with the comparability of model results. It further avoids the free use of storage energy endowment, meaning that the model could prefer to start with a higher and end with a lower storage level to save costs.

#### Detecting unintended storage cycling occurrence

USC can be caused by charging and discharging at the same time or across space and time. In this paper, we focus on the detection of USC in form of simultaneous charging and discharging that can be identified by analysing the storage operation patterns.

A straightforward approach to detect USC is to count the occurrence of simultaneous charging and discharging over the optimization horizon, which we use in later parts of the study. Here, USC may occur under three cases in energy systems with energy storage^3^: During effective charging, effective discharging, or in an idle energy state with effective net-zero charging.

The storage charging power $h_{i,s,t}^{\text{+}}$ describes the power provision from the grid to the charging component. If reduced by the charging efficiency $\eta_{i,s,+}$, it results in storage charging power $h_{i,s,t,store}^{\text{+}}$ that increases the storage energy level over time.

(Equation 13) $$h_{i,s,t,store}^{\text{+}}=\eta_{i,s,+}\cdot h_{i,s,t}^{\text{+}}$$

Likewise, store discharging power $h_{i,s,t,store}^{-}$ describes the power provision from the storage that reduces the storage energy level over time. If reduced by the discharging efficiency $\eta_{i,s,-}$, it results in the storage discharging power $h_{i,s,t}^{\text{−}}$ that provides power to the grid.

(Equation 14) $$h_{i,s,t,store}^{\text{−}}=\frac{h_{i,s,t}^{\text{−}}}{\eta_{i,s,-}}$$

The first case USC occurs under effective charging $USC_{i,s,t}^{\text{+}}$, which increases the storage energy level over time:

(Equation 15) $$\begin{array}{l} if\;h_{i,s,t,store}^{\text{+}}>h_{i,s,t,store}^{\text{−}}\;and\;h_{i,s,t,store}^{\text{−}}>0\text{:}\\ \;USC_{i,s,t}^{\text{+}}=true \end{array}$$

The second case occurs under effective discharging $USC_{i,s,t}^{\text{+}}$, which decreases the storage energy level over time:

(Equation 16) $$\begin{array}{l} if\;h_{i,s,t,store}^{\text{+}}<h_{i,s,t,store}^{\text{−}}\;and\;h_{i,s,t,store}^{\text{+}}>0\text{:}\\ \;USC_{i,s,t}^{\text{−}}=true \end{array}$$

The third case appears under non-zero equal charging and discharging $USC_{i,s,t}^{\text{=}}$, or idle energy state, which keeps the storage energy level over time constant (neglecting standing losses):

(Equation 17) $$\begin{array}{l} if\;h_{i,s,t,store}^{\text{+}}=h_{i,s,t,store}^{\text{−}}\;and\;h_{i,s,t,store}^{\text{+}}>0\text{:}\\ \;USC_{i,s,t}^{\text{=}}=true \end{array}$$

#### Numerical implementation and data

We use a stylized parameterization of PyPSA-Eur^35^ as a numerical implementation for the model to explore the occurrence and amplitude of USC, as defined in Section 4. A complete model formulation of PyPSA-Eur is provided in the Appendix of.^2^ PyPSA-Eur is a European power system model, representative of energy models that abstain from binding renewable energy targets. We apply the model to a stylised setting parameterised to the German power sector for a 100% GHG emission reduction scenario (Figure 3). We limit the available set of technologies to solar PV, onshore wind, offshore wind, as well as an H2 storage system consisting of an electrolyser, a tank, and a fuel cell. We set the spatial resolution to 16 nodes within Germany. Offshore wind power plants may be connected via high voltage alternative current (HVAC) or, in the case of sites far offshore, more costly high voltage direct current (HVDC) transmission lines. The model has perfect foresight and optimizes with an hourly temporal resolution. Weather and load data stem from 2013. Hourly load data originates from the ENTSO-E Transparency platform and are distributed across the regions depending on NUTS3 based GDP data (see more in^35^). All renewables and energy storage technologies are greenfield optimized, i.e., without considering the existing capital stock. State of charge of energy storage capacities is constrained to start and end with 100%. The self-consumption of the H2 storage tank is assumed to be zero. The transmission network is based on the network topology from 2020, considering also planned lines until 2030 from the ENTSO-E Ten Year Network Development Plan (TYNDP) 2018.^36^ Grid expansion is endogenous but limited to additional 25% newly built lines for the modeled target year to represent political hurdles of transmission expansion.^34^ Table 1 lists relevant techno-economic assumptions. This stylised setting allows for demonstrating USC in the context of energy models without binding renewable energy targets and how it can be removed by a deliberate setting of variable costs.

**Table 1:** Model input assumptions

Technology^a^^c^	Investment [€/kW]	Fixed O&M [€/kW/a]	variable cost^b^ [€/MWh]	Lifetime [a]	Efficiency [-]	Source
onshore wind	1040	25	variale	30	1	DEA^26^
offshore wind (HVAC connected)	1890	44	variable	30	1	DEA^26^
offshore (HVDC connected)	2040	47	variable	30	1	DEA^26^
PV	600	25	variable	25	1	Schröder et al.^27^
hydrogen electrolyzer	350	14	variable	25	0.8	Budischak et al.^28^
hydrogen storage tank	8.44 €/MWh	–	variable	20	1	Budischak et al.^28^
hydrogen fuel cell	339	10	variable	20	0.58	Budischak et al.^28^
transmission (submarine)	2000 €/MWkm	2%/a	0	40	1	Hagspiel et al.^29^
transmission (overhead)	400 €/MWkm	2%/a	0	40	1	Hagspiel et al.^29^

a All technologies include a discount rate of 7%.

b “Variable” means set according to scenarios.

c Unconstrained energy storage sizing and not fixed to specific energy to power ratio.

#### Experimental setup

To investigate the suggested method for removing USC, in the base case scenario we set the variable cost (EUR/MWh) of the renewable generators, H2 electrolysers, H2 tanks, and H2 fuel cells to zero. We then define further scenarios varying *ceteris paribus* the variable cost of one of these system components in the range $e\in \left\{0,10^{-5},10^{-4},10^{-3},10^{-2},10^{-1},10^{0},10^{1},10^{2},10^{3}\right\}$. That is, in each scenario, the variable cost of one system component changes according to range e, while the others are kept constant at zero. Note that, in the respective scenarios, variable costs of all renewable generators are varied at once. Cost additives of 100 or 1000 €/MWh (10 or 100 ct/kWh) represent a demonstrative, non-realistic value for all included technologies that could be interpreted as falsely set variable costs.

The double-precision arithmetic limits the amount of numbers a computer can recognise. While optimization solvers are also influenced by double-precision arithmetic, they additionally include tolerances to solve problems faster, which comes at the cost of accuracy. We vary the precision of two Gurobi solver parameters simultaneously yielding three scenarios: low, medium, and high accuracy. The first modified Gurobi parameter that impacts the precision is the FeasibilityTol or primal feasibility tolerance, which requires all constraints to satisfy a specific tolerance to be feasible.^37^ For instance, constraints such as (a∗x)<=b require to hold (a∗x)−b<=FeasibilityTol, thus expanding the solution space. We vary this value by $e\in \left\{10^{-5},10^{-6},10^{-7}\right\}$ from low to high accuracy. Simultaneously, we vary the Gurobi parameter BarConvTol, which describes the barrier solver (also known as interior point method (IPM)) termination tolerance as relative difference between the primal and dual objective values.^37^ Given one solution space for an optimization problem, this relative difference is also known as duality gap,^38^ which IPM reduce iteratively toward zero before the termination tolerance is reached. We vary this value by $e\in \left\{10^{-4},10^{-5},10^{-6}\right\}$, again, from low to high accuracy.

The computations to generate all results (10∗4∗3=120 scenarios) required 25.5 h for 1 CPU core with 8 GB memory.

## Nomenclature

Abbreviations

CSP Concentrated solar power
FLH Full load hours (h)
GHG Greenhouse gas
USC Unintended storage cycling
VRE Variable renewable energy sources

Subscripts

i Location
l Line number
r Generator technology
s Storage technology
t Time step
Variables
$H^{+}$ Storage charge capacity (MW)
$h^{+}$ Storage charge (MWh)
$H^{-}$ Storage discharge capacity (MW)
$h^{-}$ Storage discharge (MWh)
$H_{i,s}^{store}$ Store capacity (MWh)
$\bar{T}$ Energy to discharging power ratio (MWh/MW)
e Stored energy (MWh)
F Transmission line capacity (MW)
G Generator capacity (MW)
g Generated energy (MWh)
Parameters
η Efficiency
c Specific investment cost (€/MW)
o Variable cost (€/MWh)
T Optimization period
w Weighted duration

## Data Availability

Code and data to reproduce results and illustrations are available at https://github.com/pz-max/unintended-storage-cycling.
